# Mania Following Ether

**Published:** 1890-11

**Authors:** 


### Mania Following Ether.

Dr. Gorton, Superintendent of the Butler Hospital for the Insane, publishes a history of two cases. One was the case of a boy fourteen years of age who took ether before having teeth extracted. He had an attack of subacute mania followed by dementia. The second was the case of a young lady who took ether to have a number of teeth extracted. The admistration was tedious and she was several hours recovering from the influence of the anæsthetic. In a day or two she returned to her work, but her friends noticed there was a marked change in her mental condition. She afterward became insane. The patient was improving at the time of the report. Dr. C. L. Dana has stated that several of his neurasthentic patients have dated the beginning of their nervous troubles to the administration of ether.—*Am. Jour. of Insanity.*

